# Supplementary material for: Evaluating normative representation learning in generative AI for robust anomaly detection in brain imaging
Source: Nat Commun. 2025 Feb 13;16:1624. doi: 10.1038/s41467-025-56321-y (PMC11825664; doi:10.1038/s41467-025-56321-y)
Supplement: Supplementary file 1 — Supplementary Information [file 41467_2025_56321_MOESM1_ESM.pdf]

Evaluating Normative Representation Learning in Generative AI for Robust Anomaly Detection in Brain Imaging

Cosmin I. Bercea[1,2*], Benedikt Wiestler[1,3], Daniel Rueckert[1,3,4], Julia A. Schnabel[1,2,5]

[1]Technical University of Munich, Munich, Germany.
[2]Helmholtz AI and Helmholtz Center Munich, Munich, Germany.
[3]Dept. of Neuroradiology, Klinikum Rechts der Isar, Munich, Germany.
[4]Imperial College London, London, UK.
[5]King's College London, London, UK.

*Corresponding author(s). E-mail(s): cosmin.bercea@tum.de;

Supplementary information.

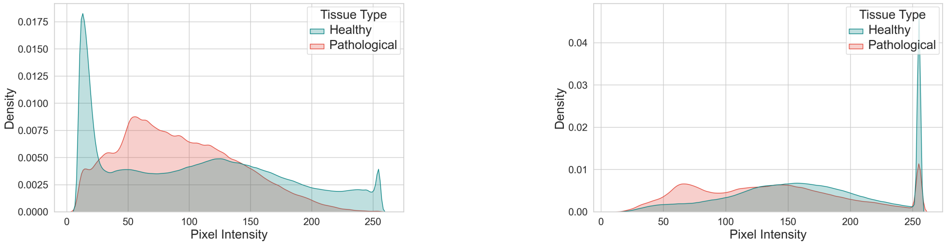

(a) Atlas Dataset.

(b) FastMRI Dataset.

Supplementary Fig. 1: Extended Figure on Pixel Intensity Distribution Comparison. This figure reveals a substantial overlap between the pixel intensity distributions of healthy (teal) and pathological (red) tissues, illustrating that our evaluation goes beyond mere intensity-based anomalies. The substantial overlap suggests that simple thresholding methods would likely be ineffective for distinguishing between these two tissue types, emphasizing the need for more sophisticated diagnostic techniques.

Supplementary Table 1: Anomaly Detection Peformance on FastMRI+. The models are assessed based on the number of detections out of the total number of samples (/N) and F1 scores for various pathologies: absent septum pellucidum (ASP), craniatomy (Cran.), dural thickening (DT), edema, encephalomalacia (Enc.), enlarged ventricles (EV) intraventricular substance (IvS), lesions (Les.), post-treatment changes (Post.), resections (Res.), sinus opacification (Sinus), white matter lesions (WML) and mass. Best results are shown in **bold**.

Method	ASP		Cran.		DT		Edema		Enc.		EV		IvS		Les.		Post.		Res.		Sinus		WML		Mass	
	/1	F1 ↑	/15	F1 ↑	/7	F1 ↑	/18	F1 ↑	/1	F1 ↑	/19	F1 ↑	/1	F1 ↑	/22	F1 ↑	/44	F1 ↑	/10	F1 ↑	/2	F1 ↑	/5	F1 ↑	/26	F1 ↑
AE [1]	0	0.00	5	6.26	2	4.76	0	0.00	0	0.00	0	0.00	0	0.00	1	2.27	14	3.81	1	0.95	**2**	8.76	0	0.00	0	0.00
VAE [2]	0	0.00	12	14.66	4	20.83	2	4.07	0	0.00	7	11.81	**1**	15.38	9	4.90	29	14.96	8	16.13	**2**	16.67	0	0.00	16	13.37
LTM [3]	0	0.00	13	18.75	5	29.75	4	11.48	**1**	22.22	16	44.75	**1**	15.38	10	6.07	30	11.76	8	16.87	**2**	14.22	1	1.02	19	16.97
f-AnoGAN [4]	0	0.00	**14**	19.19	3	9.54	2	3.44	0	0.00	13	22.82	**1**	12.50	8	3.68	30	12.08	8	17.78	**2**	9.16	2	1.58	16	12.36
SI-VAE [5]	0	0.00	11	14.47	4	19.31	0	0.00	0	0.00	9	15.70	**1**	18.18	6	3.97	27	9.44	8	24.55	2	11.42	2	6.03	12	7.08
RA [6]	**1**	15.38	13	34.78	6	**52.65**	12	45.56	**1**	66.67	18	77.54	**1**	50.00	17	29.50	35	30.78	**10**	**54.32**	**2**	**26.67**	**5**	15.50	21	30.78
DDPM-G [7]	0	0.00	**14**	16.86	**7**	47.02	5	9.07	**1**	28.57	12	22.70	**1**	13.33	11	5.32	34	14.32	8	17.33	**2**	11.26	2	2.87	17	12.01
DDPM-S [7]	1	14.29	9	14.04	6	38.48	14	35.51	**1**	40.00	17	51.23	**1**	28.57	16	16.92	32	15.94	**10**	33.21	1	1.72	3	8.14	22	12.42
ceVAE [8]	0	0.00	**14**	16.99	4	22.70	3	4.52	0	0.00	4	5.91	**1**	20.00	10	5.05	32	14.76	7	15.00	**2**	15.88	0	0.00	19	14.12
MorphAEus [9]	0	0.00	13	17.10	6	37.85	9	17.38	**1**	40.00	10	15.77	**1**	28.57	15	10.94	35	15.51	**10**	23.47	2	11.54	**2**	3.02	22	17.24
MAE [10]	0	0.00	12	18.48	3	8.81	2	4.63	0	0.00	7	17.37	**1**	15.38	7	3.71	29	14.55	7	24.23	**2**	15.11	0	0.00	14	13.66
pDDPM [11]	**1**	16.67	12	20.39	**7**	50.87	**17**	**49.46**	**1**	66.67	13	27.56	**1**	33.33	19	21.22	**40**	18.67	**10**	37.86	**2**	5.35	**5**	17.70	25	29.11
PHANES [12]	**1**	18.18	**14**	36.15	**7**	51.31	16	43.28	**1**	**100.00**	**19**	**80.51**	1	40.00	**21**	27.31	**40**	29.65	**10**	49.33	1	10.00	2	4.07	24	33.89
autoDDPM [13]	**1**	**25.00**	13	**37.03**	6	50.27	16	45.78	**1**	**66.67**	18	40.84	1	66.67	**21**	**36.30**	**40**	**38.97**	**10**	49.44	1	14.29	**5**	**22.16**	**26**	**49.57**

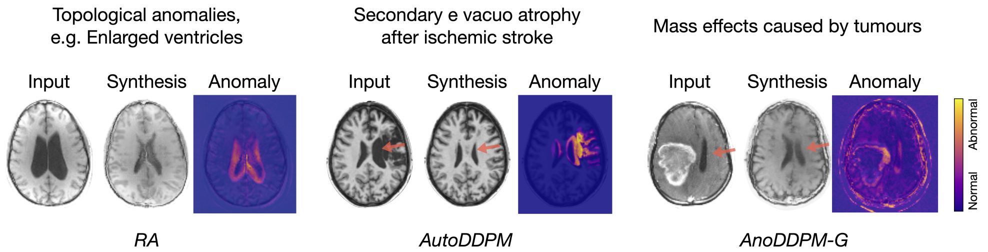

Supplementary Fig. 2: Extended Figure on the Broad Anomaly Detection Task. Our evaluation goes beyond mere intensity-based anomalies, showcasing the application of three different anomaly detection techniques on MRI brain scans. These techniques excel in identifying and visualizing a variety of structural and topological anomalies. This includes atrophy, such as enlarged ventricles, changes following ischemic strokes, and mass effects due to tumors.

Supplementary Table 2: Comparison of Brain MRI Datasets: Demographics, Imaging Characteristics, Disease Types, Annotations, Preprocessing, and Acquisition Types.

**Characteristic**	**IXI**	**fastMRI+**	**ATLAS**
Number of Subjects	581	5847	1271
Number of Annotated Scans	581	1001	655
Age Range (years)	20–86	Not specified	Not shared
Sex Distribution	Male = 277, Female = 342	Not specified	Not shared
Scanner Vendors	Philips, GE, Siemens	11 different scanners	Not specified
Scanning Location	3 hospitals in London, UK	5 clinical locations	Not specified
Field Strength (T)	1.5, 3.0	1.5, 3.0	1.5, 3.0
Image Sequences	**T1**, T2, PD, MRA, DWI	**Axial T1**, Axial T2, Axial FLAIR	**T1**
Disease Type	Healthy volunteers	Various pathologies	Ischemic stroke lesions
Annotation Type	None	Bounding box annotations	Pixel-wise segmentation
Preprocessing	Not specified	Cropped for de-identification	Intensity standardization<br>Linear registration to MNI 152<br>Defacing
Acquisition Type	3D	2D Axial acquisition	3D

References

[1] Baur, C., Denner, S., Wiestler, B., Navab, N. & Albarqouni, S. Autoencoders for unsupervised anomaly segmentation in brain mr images: A comparative study. *Medical Image Analysis* 101952 (2021).

[2] Zimmerer, D., Isensee, F., Petersen, J., Kohl, S. & Maier-Hein, K. Unsupervised anomaly localization using variational auto-encoders. *Medical Image Computing and Computer Assisted Intervention* 289–297 (2019).

[3] Pinaya, W. H. *et al.* Unsupervised brain imaging 3d anomaly detection and segmentation with transformers. *Medical Image Analysis* **79**, 102475 (2022).

[4] Schlegl, T., Seeböck, P., Waldstein, S. M., Langs, G. & Schmidt-Erfurth, U. f-AnoGAN: Fast unsupervised anomaly detection with generative adversarial networks. *Medical Image Analysis* **54**, 30–44 (2019).

[5] Daniel, T. & Tamar, A. Soft-IntroVAE: Analyzing and improving the introspective variational autoencoder. *Proceedings of the IEEE/CVF Conference on Computer Vision and Pattern Recognition* 4391–4400 (2021).

[6] Bercea, C. I., Wiestler, B., Rueckert, D. & A, S. J. Generalizing unsupervised anomaly detection: Towards unbiased pathology screening. *International Conference on Medical Imaging with Deep Learning* (2023).

[7] Wyatt, J., Leach, A., Schmon, S. M. & Willcocks, C. G. Anoddpm: Anomaly detection with denoising diffusion probabilistic models using simplex noise. *Proceedings of the IEEE/CVF Conference on Computer Vision and Pattern Recognition Workshops* 650–656 (2022).

[8] Zimmerer, D., Kohl, S. A., Petersen, J., Isensee, F. & Maier-Hein, K. H. Context-encoding variational autoencoder for unsupervised anomaly detection. *arXiv preprint arXiv:1812.05941* (2018).

[9] Bercea, C. I., Rueckert, D. & Schnabel, J. A. What do AEs learn? Challenging Common Assumptions in Unsupervised Anomaly Detection. *Medical Image Computing and Computer-Assisted Intervention* 304–314 (2023).

[10] He, K. *et al.* Masked autoencoders are scalable vision learners. *Proceedings of the IEEE/CVF conference on computer vision and pattern recognition* 16000–16009 (2022).

[11] Behrendt, F., Bhattacharya, D., Krüger, J., Opfer, R. & Schlaefer, A. Patched diffusion models for unsupervised anomaly detection in brain mri. *International Conference on Medical Imaging with Deep Learning* (2023).

[12] Bercea, C. I., Wiestler, B., Rueckert, D. & Schnabel, J. A. Reversing the abnormal: Pseudo-healthy generative networks for anomaly detection. *Medical Image*

Computing and Computer-Assisted Intervention (2023).

[13] Bercea, C. I., Neumayr, M., Rueckert, D. & Schnabel, J. A. Mask, stitch, and re-sample: Enhancing robustness and generalizability in anomaly detection through automatic diffusion models. *ICML 3rd Workshop on Interpretable Machine Learning in Healthcare* (2023).
