# Supplementary material for: Evaluating normative representation learning in generative AI for robust anomaly detection in brain imaging
Source: Nat Commun. 2025 Feb 13;16:1624. doi: 10.1038/s41467-025-56321-y (PMC11825664; doi:10.1038/s41467-025-56321-y)
Supplement: Supplementary file 2 — Reporting Summary [file 41467_2025_56321_MOESM2_ESM.pdf]

nature portfolio

Corresponding author(s): Cosmin I. Bercea
Last updated by author(s): Nov 23, 2024

Reporting Summary

Nature Portfolio wishes to improve the reproducibility of the work that we publish. This form provides structure for consistency and transparency in reporting. For further information on Nature Portfolio policies, see our Editorial Policies and the Editorial Policy Checklist.

Statistics

For all statistical analyses, confirm that the following items are present in the figure legend, table legend, main text, or Methods section.

n/a	Confirmed	
☐	☒	The exact sample size (*n*) for each experimental group/condition, given as a discrete number and unit of measurement
☐	☒	A statement on whether measurements were taken from distinct samples or whether the same sample was measured repeatedly
☒	☐	The statistical test(s) used AND whether they are one- or two-sided<br>*Only common tests should be described solely by name; describe more complex techniques in the Methods section.*
☒	☐	A description of all covariates tested
☐	☒	A description of any assumptions or corrections, such as tests of normality and adjustment for multiple comparisons
☐	☒	A full description of the statistical parameters including central tendency (e.g. means) or other basic estimates (e.g. regression coefficient) AND variation (e.g. standard deviation) or associated estimates of uncertainty (e.g. confidence intervals)
☒	☐	For null hypothesis testing, the test statistic (e.g. *F*, *t*, *r*) with confidence intervals, effect sizes, degrees of freedom and *P* value noted<br>*Give P values as exact values whenever suitable.*
☒	☐	For Bayesian analysis, information on the choice of priors and Markov chain Monte Carlo settings
☒	☐	For hierarchical and complex designs, identification of the appropriate level for tests and full reporting of outcomes
☒	☐	Estimates of effect sizes (e.g. Cohen's *d*, Pearson's *r*), indicating how they were calculated

Our web collection on statistics for biologists contains articles on many of the points above.

Software and code

Policy information about availability of computer code

Data collection	Since our study exclusively utilized public datasets and did not involve the collection of any new data.
Data analysis	We made the code publicly available at: https://github.com/ci-ber/GenAI_UAD

For manuscripts utilizing custom algorithms or software that are central to the research but not yet described in published literature, software must be made available to editors and reviewers. We strongly encourage code deposition in a community repository (e.g. GitHub). See the Nature Portfolio guidelines for submitting code & software for further information.

Data

Policy information about availability of data

All manuscripts must include a data availability statement. This statement should provide the following information, where applicable:

- Accession codes, unique identifiers, or web links for publicly available datasets
- A description of any restrictions on data availability
- For clinical datasets or third party data, please ensure that the statement adheres to our policy

IXI: https://brain-development.org/ixi-dataset/; FastMRI: https://fastmri.med.nyu.edu; Fast MRI + Labels: https://github.com/microsoft/fastmri-plus; Atlas: https://fcon_1000.projects.nitrc.org/indi/retro/atlas.html

Research involving human participants, their data, or biological material

Policy information about studies with human participants or human data. See also policy information about sex, gender (identity/presentation), and sexual orientation and race, ethnicity and racism.

Reporting on sex and gender	In our study, the primary objective was to assess the general performance of generative models across a broad population. Our focus was to understand and evaluate the overall capabilities and limitations of these models in a wide-ranging context. It is important to note that our study did not specifically delve into subgroup analyses or examine potential biases related to sex, gender, race, or age groups. The rationale behind this approach was to maintain a broad perspective and avoid narrowing our findings to specific subgroups in the initial stages of this research. We acknowledge the significance and impact of sex-and-gender-based analysis in research and fully intend to explore these aspects in future work.
Reporting on race, ethnicity, or other socially relevant groupings	Our study did not specifically focus on race, ethnicity, or other socially relevant groupings. The primary aim was to assess the performance of generative models across a general population, without delving into subgroup analyses. This approach was chosen to maintain a broad perspective in the initial research phase. Future studies may explore these aspects in more detail.
Population characteristics	We did not report specific population characteristics in our manuscript due to the reasons previously mentioned, including the broad scope of the study and to maintain conciseness in the manuscript. However, all relevant information about the population characteristics of the public datasets used is available online for reference
Recruitment	Since our study exclusively utilized public datasets and did not involve the collection of any new data, issues of recruitment and ethics oversight pertaining to data collection were not directly applicable in this context.
Ethics oversight	*Identify the organization(s) that approved the study protocol.*

Note that full information on the approval of the study protocol must also be provided in the manuscript.

Field-specific reporting

Please select the one below that is the best fit for your research. If you are not sure, read the appropriate sections before making your selection.

☒ Life sciences ☐ Behavioural & social sciences ☐ Ecological, evolutionary & environmental sciences

For a reference copy of the document with all sections, see nature.com/documents/nr-reporting-summary-flat.pdf

Life sciences study design

All studies must disclose on these points even when the disclosure is negative.

Sample size	We used a total of 1,348 samples for training and testing, which came from three different datasets.
Data exclusions	We excluded a total of 230 samples from the Atlas dataset. This included 210 images without pathologies from the testing set, and 20 samples that had visual artifacts which were not annotated.
Replication	We have made the code used in our study, including the implementation of all methods, evaluation metrics, and used random seeds, freely available online within our deep learning framework. This is to facilitate replication and encourage further research in the field.
Randomization	We utilized images with no known pathologies for training, randomly split into train, validation, and test sets, ensuring no patient overlap. The pathological datasets were reserved for the downstream anomaly detection task to test the model's efficacy in identifying anomalies.
Blinding	When performing the random split of data for training, validation, and testing, we did not access any sensitive information, ensuring a blind allocation process.

Reporting for specific materials, systems and methods

We require information from authors about some types of materials, experimental systems and methods used in many studies. Here, indicate whether each material, system or method listed is relevant to your study. If you are not sure if a list item applies to your research, read the appropriate section before selecting a response.

Materials & experimental systems

n/a	Involved in the study
☒	☐ Antibodies
☒	☐ Eukaryotic cell lines
☒	☐ Palaeontology and archaeology
☒	☐ Animals and other organisms
☒	☐ Clinical data
☒	☐ Dual use research of concern
☒	☐ Plants

Methods

n/a	Involved in the study
☒	☐ ChIP-seq
☒	☐ Flow cytometry
☒	☐ MRI-based neuroimaging

Plants

Seed stocks	*Report on the source of all seed stocks or other plant material used. If applicable, state the seed stock centre and catalogue number. If plant specimens were collected from the field, describe the collection location, date and sampling procedures.*
Novel plant genotypes	*Describe the methods by which all novel plant genotypes were produced. This includes those generated by transgenic approaches, gene editing, chemical/radiation-based mutagenesis and hybridization. For transgenic lines, describe the transformation method, the number of independent lines analyzed and the generation upon which experiments were performed. For gene-edited lines, describe the editor used, the endogenous sequence targeted for editing, the targeting guide RNA sequence (if applicable) and how the editor was applied.*
Authentication	*Describe any authentication procedures for each seed stock used or novel genotype generated. Describe any experiments used to assess the effect of a mutation and, where applicable, how potential secondary effects (e.g. second site T-DNA insertions, mosiacism, off-target gene editing) were examined.*
